# The Effect of Probiotics on Childhood Constipation: A Randomized Controlled Double Blind Clinical Trial

**DOI:** 10.1155/2014/937212

**Published:** 2014-04-09

**Authors:** M. Sadeghzadeh, A. Rabieefar, P. Khoshnevisasl, N. Mousavinasab, K. Eftekhari

**Affiliations:** ^1^Department of Pediatrics, Zanjan Metabolic Disease Research Center, Zanjan University of Medical Sciences, Zanjan, Iran; ^2^Zanjan University of Medical Sciences, Zanjan, Iran; ^3^Department of Pediatrics, Social Determinants of Health Research Center, Zanjan University of Medical Sciences, Zanjan, Iran; ^4^Department of Epidemiology, Zanjan University of Medical Sciences, Zanjan, Iran; ^5^Department of Pediatrics, Zanjan University of Medical Sciences, Zanjan, Iran

## Abstract

*Background*. Inconsistent data exist about the role of probiotics in the treatment of constipated children. The aim of this study was to investigate the effectiveness of probiotics in childhood constipation. * Materials and Methods*. In this placebo controlled trial, fifty-six children aged 4–12 years with constipation received randomly lactulose plus Protexin or lactulose plus placebo daily for four weeks. Stool frequency and consistency, abdominal pain, fecal incontinence, and weight gain were studied at the beginning, after the first week, and at the end of the 4th week in both groups. * Results*. Forty-eight patients completed the study. At the end of the fourth week, the frequency and consistency of defecation improved significantly (*P* = 0.042 and *P* = 0.049, resp.). At the end of the first week, fecal incontinence and abdominal pain improved significantly in intervention group (*P* = 0.030 and *P* = 0.017, resp.) but, at the end of the fourth week, this difference was not significant (*P* = 0.125 and *P* = 0.161, resp.). A significant weight gain was observed at the end of the 1st week in the treatment group. * Conclusion*. This study showed that probiotics had a positive role in increasing the frequency and improving the consistency at the end of 4th week.

## 1. Introduction

Constipation is a common disorder in children and could have a destructive effect on the physical as well as psychological aspects of health [1]. Its prevalence varies from 0.07% to 29.6% in different studies [2]. Organic causes cannot be found in more than 90% of cases [3]. Constipation is defined as the painful passage of stool less than twice a week or less than once every three days [4]. Usual treatments including toilet training, family education, dietary changes, and the use of laxatives, although useful, are not completely satisfactory [1, 5]. Therefore, there is a growing interest to find a new solution [3].

Currently, probiotics were used as an adjunctive treatment for a lot of childhood diseases. The role of probiotics in gastrointestinal diseases as well as allergic disorders, atopic dermatitis, prevention of infections, necrotizing enterocolitis, and infantile colic was shown in many studies [5–8].

It seems that probiotics, which are live microbial ingredients, produce lactic and acetic acids and influence the peristalsis of intestines by reducing colonic pH [3, 5].

Although the role of probiotics is well studied in adults, there were few data on its effectiveness in childhood constipation with contradictory results [1, 3, 9, 10]. Therefore, we conducted this study to evaluate the role of probiotics on our constipated patients.

## 2. Materials and Methods

This randomized double blind controlled study was conducted on 56 children 4–12 years old with chronic constipation who were referred to Ayatollah Moussavi hospital clinics in Zanjan, Iran, from October 2011 to March 2012. All children fulfilled Rome III criteria for chronic constipation. The exclusion criteria consisted of any underlying diseases, history of hospital admission, or any gastrointestinal or nutritional problems other than constipation. This study was approved by the Medical Ethics Committee of Zanjan University of Medical Sciences (Ethical code: 904295). Informed written consent was signed by parents of all patients before any intervention.

The sample size was calculated by the formula *n* = [*Z*_1−*α*/2_+*Z*_1−*β*_]^2^[*P*_1_(1 − *P*_1_) + *P*_2_(1 − *P*_2_)]/(*P*_1_ − *P*_2_)^2^ based on *P*_1_ = 89%, *P*_2_ = 56%, *α* = 0/05, 1 − *β* = 0/80, *z* = 1/95, and 0/84, respectively (*n* = 56).

The patients were randomly allocated into two groups who received lactulose (1 mL/kg/d) plus Protexin (Nikooteb Company, Tehran, Iran) one sachet daily or lactulose plus placebo alone for four month. The control group was matched according to sex and age.

A period of one week was estimated as a wash-out period for those who used any drugs for their constipation. Each sachet of Protexin was composed of seven probiotic bacteria including* Lactobacillus casei* PXN 37,* Lactobacillus rhamnosus* PXN 54,* Streptococcus thermophiles* PXN 66,* Bifidobacterium breve* PXN 25,* Lactobacillus acidophilus* PXN 35,* Bifidobacterium infantis (child specific)* PXN 27, and* Lactobacillus bulgaricus* PXN 39, TVC: 1 billion CFU TVC: 1 × 10^9^. The placebo was supplied by Nikooteb company, the provider of probiotics in Iran, as innocent powder in identical sachets and stored in a cool and dry place until use.

Each patient was visited by the researcher and completely evaluated for organic diseases, and a questionnaire including demographic data, past medical history, drug history, symptoms of constipation, and physical examination was completed before the study. The patients with comorbid conditions were excluded from the study.

After the first and fourth weeks of intervention, a second questionnaire was completed for symptoms of constipation including the frequency of defecation, stool consistency, abdominal pain, frequency of fecal incontinence, and side effects in both groups. Fecal frequency, consistency (hard, normal, and soft), and weight gain of all patients were recorded. Fecal incontinence and abdominal pain were looked for only in patients who had these symptoms before the intervention.

Data were analyzed using SPSS software Version 16.0. Number of bowel movements and fecal incontinence episodes in baseline information were analyzed by Freidman test. The Student's *t*-test was used for parametric data and chi-square analysis was used for categorical measures. *P* value of <0.05 was considered significant.

## 3. Results

A total of 56 patients were enrolled in the study. Four patients in the intervention group (three in the first week and one in the fourth week) refused to complete the study and were excluded. Four patients in the control group had not completed the study as well (two did not refer for follow-up and two patients did not fulfill criteria Rome III during the study), and were excluded. At the end, two groups of 24 patients were studied.

In the intervention group, 14 males (58.3%) and 10 females (41.7%) completed the study. The control group consisted of 10 males (41.7%) with 14 females (58.3%). The difference of the two groups was not statistically significant (*P* = 0.248). The mean age of patients in treatment group was 6.1 ± 2.4 and in control group was 6.3 ± 1.9(*P* = 0.739). The demographic data are shown in Table 1.

In the intervention group, 54.2% and in controls 37.5% had fecal incontinence before the intervention (*P* = 0.247). In the first group, 66.7% had abdominal pain in the beginning of the study compared to 58.3% in the second group (*P* = 0.551).  These patients were followed for improvement of their symptoms till 4th week.

As shown in Table 2, at the end of the fourth week, the frequency and consistency of defecation improved significantly (*P* = 0.042, *P* = 0.049, resp.).

At the end of the first week, fecal incontinence and abdominal pain improved significantly in intervention group (*P* = 0.030, *P* = 0.017, resp.) but, at the end of the fourth week, this difference was not significant (*P* = 0.125, *P* = 0.161, resp.) (Table 3).

Surprisingly, we found that, at the end of the first week, probiotics had significantly improved weight gain (more than 10%) (*P* = 0.002), and this difference, although, continued but was not significant at the end of the fourth week (*P* = 0.098).

No side effects were noted during the treatment.

## 4. Discussion

It seems that probiotics which are live microbial ingredients competitively exclude pathogenic bacteria and improve gastrointestinal upsets. By producingshort-chain fatty acids, lactic acid, and acetic acid, they reduce colonic PH, change gut microflora, and influence the peristalsis of intestines [3, 5].

Our study showed that probiotics were significantly effective in improving the stool frequency and consistency in intervention group at the end of the 4th week. A significant decrease in fecal incontinence and abdominal pain and increasing body weight were found by the end of the first week in treatment group which was not significant at the end of the 4th week.  There are many studies with the same results [1, 3, 11–16].

The study of Saneian comparing placebo plus mineral oil and probiotics plus mineral oil on 60 patients in Isfahan, Iran, revealed that stool frequency, consistency, pain at defecation, and soiling improved significantly in intervention group [1].

The study of Bekkali on twenty 4–16-year-old children receiving probiotics revealed that, after 4 weeks, the frequency of bowl movements had been increased and a significant decrease in fecal incontinence and abdominal pain was observed [3]. These results are similar to our results.

Koebnick concluded that at the end of 4th week 89% ofconstipated patients receiving probiotics significantly improved compared to 56% of controls [11].

The study of Ardatskaia on 30 patients having irritable bowel syndrome with predominance of constipation showed that Normoflorin therapy had normalized the intestinal motor activity through changes in microbial flora of the intestines [12].

In a crossover trial conducted in Brazil by Guerra, studying 59 constipated students, after 5 weeks, the cases who received probiotic yogurt had significant improvement in defecation frequency (*P* = 0.012), defecation pain (*P* = 0.046), and abdominal pain (*P* = 0.015) compared to students who get only yogurt [13].

Jayasimhan had studied 120 adults with constipation and followed them after 7 days. He concluded that probiotics had significantly improved stool frequency and consistency [14]. These results are similar to our results at the end of the first week.

The study of Khodadad in Tehran on 102 constipated children showed that probiotic plus mineral oil increased stool frequency significantly comparing with mineral oil plus placebo and probiotic plus placebo. On the other hand, stool consistency, abdominal pain, and fecal incontinence were improved, although the difference was not significant. The results of this study were similar to our investigation but improvement of stool consistency was significant in our study [15].

In a meta-analysis by Miller and Ouwehand, probiotics had a short-term effect on reducing intestinal transit time in constipated adults. A greater effect on patients with versus without constipation and older versus younger was shown [16].

In all these studies, stool frequency has been improved which could be due to change in intestinal flora, although few studies have reevaluated gut microflora [12]. Although the differences in improvement of various symptoms could be due to regimens used by patients, the mixture of pre- and probiotics and different bacteria used can also explain these diversities. The decrease in fecal incontinence and abdominal pain and increasing body weight that was found by the end of the first week in treatment group but not significant at the end of the 4th week could be explained by the chronic nature of the disease, a better effect of this drug in short term, and the tolerance to treatment.

Conversely, there are some studies which are not similar to our findings:

Vandenplas et al. stated that probiotics had limited role in controlling the constipation, although its role in antibiotic-associated diarrhea and acute gastroenteritis was confirmed [6].

Banaszkiewicz and Szajewska had studied eighty-four constipated children (2–16 years of age) receiving 1 mL/kg/day of 70% lactulose plus 10^9^ colony-forming units (CFU) of* Lactobacillus* GG (LGG) orally twice daily for 12 weeks comparing with a control group and concluded that LGG was not an effective adjunct to lactulose in children with constipation [17]. We have used lactulose in our patients too, because it was well tolerated and easily accessible and different results may be due to the composition of probiotics used.

Mazlyn and coworkers demonstrated that adults with functional constipation did not have significant alleviation in constipation severity or stool frequency, consistency, and quantity comparing to controls after 4 weeks of treatment with probiotics [18].

The study of Tabbers et al. on 159 constipated children receiving fermented dairy product containing Bifidobacterium lactis strain showed that, in spite of improvement in stool frequency comparing to baseline, the result was not comparable to controls [19].

Considering these controversies, it seems that larger studies are needed to clarify the effect of probiotics in constipation. It is recommended to control strictly the regimens used in both groups, and it seems that a mixture of pre- and probiotics containing all useful flora would be promising.

Also, we found a significant increase in body weight which was not mentioned in other studies and it may be due to improved appetite after decreasing intestinal transit time. Further investigations are needed to prove this effect.

## 5. Conclusion

This investigation revealed significantly increased bowel frequency and improved stool consistency with the combination of lactulose and probiotics. In our study, the decrease in episodes of fecal incontinence and abdominal pain was significant compared to control group at the end of the first week that may be due to a better effect of this drug in short term.

## Figures and Tables

**Table 1:** Demographic characteristics of patients.

Variables	Intervention group	Control group	Total	*P* value
Gender				
Male	14 (58.3%)	10 (41.7%)	24 (50%)	(*P* = 0.248)
Female	10 (41.7%)	14 (58.3%)	24 (50%)	
Mean age (year)	6.1 ± 2.4	6.3 ± 1.9		(*P* = 0.739)

**Table 2:** Comparison of symptoms between the beginning and end of the 1st and 4th weeks.

Variables		Treatment (mean ± SD)	Placebo (mean ± SD)	*P* value
Stool frequency	Beginning to 1st week	1.67 ± 0.82	0.79 ± 0.83	0.042
	Beginning to 4th week	2.08 ± 0.65	1.54 ± 0.98	
	1st to 4th week	0.92 ± 0.72	0.75 ± 0.61	

Stool consistency*	Beginning to 1st week	0.42 ± 0.50	0.21 ± 0.41	0.049
	Beginning to 4th week	0.88 ± 0.45	0.63 ± 0.50	
	1st to 4th week	0.46 ± 0.51	0.42 ± 0.50	

*Stool consistency: 1: hard, 2: normal, and 3: soft.

**Table 3:** Symptom changes at the end of the 1st week.

Symptom	Treatment frequency, percent	Placebo frequency, percent	*P* value
With fecal incontinence	4 (30.8%)	7 (77.8%)	0.030
Without fecal incontinence	11 (69.2%)	2 (22.2%)	
Total (fecal incontinence)	**15 (100%)**	**9 (100%)**	
With abdominal pain	7 (43.8%)	12 (85.7%)	0.017
Without abdominal pain	9 (56.2%)	2 (14.3%)	
Total (abdominal pain)	**16 (100%)**	**14 (100%)**	
With weight gain	10 (41.7%)	1 (4.2%)	0.002
Without weight gain	14 (58.3%)	23 (95.8)	
Total weight gain	**24 (100%)**	**24 (100%)**	
